# Aflatoxin B1 modulates mixture-induced neurotoxicity under multi-contaminant exposure: insights from combination modeling in SH-SY5Y cells

**DOI:** 10.3389/fphar.2026.1857857

**Published:** 2026-07-03

**Authors:** Helena Ramos, Ana Margarida Araújo, Isabel M. P. L. V. O. Ferreira, Miguel A. Faria

**Affiliations:** LAQV-REQUIMTE, Faculty of Pharmacy, University of Porto, Porto, Portugal

**Keywords:** acrylamide, aflatoxin B1, cadmium, chou-talalay, CISNE, combination index, mixture effects, neurotoxicity

## Abstract

**Introduction:**

The chemical exposome is increasingly implicated in neurodegenerative diseases (NDs). However, although human exposure to environmental and dietary contaminants typically occurs as complex mixtures, neurotoxicity assessments still largely focus on single compounds. This study investigated the neurotoxic interactions between aflatoxin B1 (AFB1), acrylamide (ACR), and cadmium in a neuronal cell model.

**Methods:**

SH‐SY5Y cells were exposed to AFB1, ACR, and Cd^2+^ individually or in fixed‐ratio mixtures for 72 h. Mixture interactions were evaluated from MTT data using the combination index (CI) approach implemented through the Chou‐Talalay (CT) and CISNE frameworks.

**Results:**

Individual potency ranked Cd^2+^>AFB1>ACR. At low effect levels, AFB1‐containing mixtures showed relevant deviations from additivity. The ACR/AFB1 combination displayed synergism at IC10 (CISNE CI 0.62), with concentrations reducing to 4.4‐fold for ACR and 2.6‐fold for AFB1. AFB1/Cd^2+^ interactions were model-dependent, with CT indicating synergism at IC10 (CI 0.43), whereas CISNE indicated near additivity. ACR/Cd^2+^ and ternary mixtures displayed strong antagonism at low effect levels (CISNE CI 7.07 at IC10). Interactions interpretation was generally consistent across models, but CT amplified deviations from additivity at low levels.

**Conclusion:**

Overall, these findings identify AFB1 as a key contributor to mixture‐induced neurotoxicity and support the use of CISNE modelling to improve low‐effect interaction analysis in food contaminant risk assessment.

## 1 Introduction

The chemical exposome is a central unresolved challenge in toxicology and public health, being increasingly recognized as a major determinant of chronic disease risk (Crepet et al., 2025). Neurotoxicity is especially relevant within the exposome framework considering that neurons are long-lived, metabolically active cells with limited regenerative capacity, which makes them especially vulnerable to cumulative cellular stress over time, namely, to food-borne and environmental contaminants such as mycotoxins, processing-related contaminants, and heavy metals (Cannon and Timothy Greenamyre, 2011; Kisby and Spencer, 2011; Min and Min, 2016; Liu et al., 2017; Pandics et al., 2023; Song et al., 2024). Exposure to complex mixtures of environmental and dietary chemicals over a prolonged period may interact with genetic susceptibility and age-related biological vulnerability, thereby contributing to the onset and/or progression of neurodegenerative diseases (NDs), including Alzheimer’s disease (AD) and Parkinson’s disease (PD) (Sakowski et al., 2024). Proposed convergent mechanisms linking chemical exposure and neurodegeneration include oxidative stress, mitochondrial dysfunction, neuroinflammation, and exposure-associated epigenetic alterations, supporting the hypothesis that cumulative toxic insults contribute to ageing-related brain vulnerability (Nabi and Tabassum, 2022; Lefevre-Arbogast et al., 2024a; Sakowski et al., 2024).

Recently, the European Human Biomonitoring Initiative (HBM4EU) prioritized eighteen chemical substances or groups based on internal exposure levels, including pesticides, heavy metals, mycotoxins, and processing-related contaminants such as acrylamide (Arce-Lopez et al., 2020; Huang and Fang, 2020; Govarts et al., 2023). Within this framework, dietary exposure has been identified as a dominant and continuous contributor to the chemical exposome, highlighting food-related contaminants (FCs) as relevant contributors to long-term neurological health (Lefevre-Arbogast et al., 2024a; Lefevre-Arbogast et al., 2024b).

Among dietary contaminants, mycotoxins are particularly relevant as they contaminate widely consumed staple commodities, including cereals, nuts, and grains (Eskola et al., 2020). A 14-year analysis of mycotoxins notifications in the EU’s Rapid Alert System for Food and Feed (RASFF) identified aflatoxins as the predominant hazard, accounting for nearly 90% of all mycotoxin notifications (Jallow et al., 2021; Kow et al., 2025). Among these, aflatoxin B1 (AFB1) is the most toxicologically relevant congener. Classified as a Group 1 carcinogen by the International Agency for Research on Cancer (2012), AFB1 remains a major global food safety concern, particularly in developing countries where regulatory limits are lacking or not effectively enforced (Jallow et al., 2021). In addition to their frequent detection in staple commodities, AFB1 is resistant to typical food-processing temperatures, facilitating persistent dietary exposure (Rushing and Selim, 2019; Arce-Lopez et al., 2020). Experimental evidence indicates that AFB1 can engage neurotoxicity-relevant pathways, including oxidative stress and mitochondrial dysfunction and neuroinflammation, mechanisms closely linked to NDs (Song et al., 2024; Abia et al., 2025; Lin et al., 2025a).

Importantly, in human dietary contexts, AFB1 exposure can coincide with exposure to other contaminants, namely, acrylamide (ACR), a processing-related compound formed in carbohydrate-rich foods under high-temperature conditions, or heavy metals, such as cadmium, from environmental contamination of food crops (Mattsson, 2007; Fu et al., 2022; Bridgeman et al., 2024; Zhu et al., 2024). Although originating from different stages of the food chain, these FCs may converge in cereal-based and processed foods, supporting their relevance as a plausible dietary co-exposure scenario. Nevertheless, traditional risk assessment frameworks remain largely single-compound oriented (Bridgeman et al., 2024; Crepet et al., 2025). Studying the toxicity of chemical mixtures instead of their individual constituents is therefore essential to better approximate real-life exposure scenarios and to clarify their potential role in the development of chronic diseases, particularly those with long latency periods (Hernandez and Tsatsakis, 2017). Combined exposures can produce more-than-additive effects, commonly referred to as synergism, or less-than-additive effects, described as antagonism (Duarte and Vale, 2022). Interaction effects are not intrinsic properties of a mixture but are inferred by comparing experimentally observed responses with effects predicted by a chosen reference model. Consequently, whether a combination is classified as synergistic, antagonistic, or additive depends directly on the selected model and its underlying biological and mathematical assumptions, meaning that the same dataset may yield different interpretations depending on the reference framework applied (Foucquier and Guedj, 2015; Lasch et al., 2020). Concentration addition (CA) and independent action (IA) constitute the standard reference models for mixture effect prediction in toxicology and regulatory risk assessment (Rodea-Palomares et al., 2015; Duarte and Vale, 2022). Whilst CA assumes that mixture components behave as dilutions of one another and is most appropriate when compounds share similar modes of action and parallel concentration-response curves, in contrast, IA assumes that components act independently (Foucquier and Guedj, 2015). However, alternative quantitative frameworks such as the Chou-Talalay combination index model have been increasingly applied in food and environmental contaminant research to characterize deviations from additivity across effect levels (Kifer et al., 2020). *In vitro* models are valuable for studying contaminant interactions under controlled conditions. SH-SY5Y cells are a widely used human-derived neuronal model for investigating toxicant-induced neurotoxicity (Lopez-Suarez et al., 2022).

In the context of human dietary exposure and emerging evidence linking AFB1 to neurotoxicity-relevant pathways, we aimed to determine whether AFB1 acts as a key contributor to mixture-induced toxic effects. Specifically, this study investigated how co-exposure to chemically distinct FCs, specifically aflatoxin B1 (AFB1), acrylamide (ACR) and cadmium (Cd^2+^), influences neuronal viability in human SH-SY5Y cells following 72 h of exposure. Experimental cytotoxicity data were compared with effects predicted by the reference null model of IA, and the nature and magnitude of interactions were subsequently characterized using combination index approaches based on the Chou–Talalay and CISNE frameworks. Furthermore, we examined how the choice of the interaction model influences the classification of synergism, additivity, or antagonism, particularly at low effect levels, the most relevant to human exposure scenarios.

## 2 Materials and methods

### 2.1 Materials

Aflatoxin B1 (CAS 1162–65-8, >98%) and acrylamide (CAS 79–06-1, >99%) were purchased from Sigma Aldrich (St Louis, MO, USA). Cadmium chloride (CdCl_2_.2_1/2_H_2_O, 98%) was kindly provided by the Laboratory of Organic and Pharmaceutical Chemistry of the Faculty of Pharmacy, University of Porto. For cell culture maintenance, high glucose Dulbecco’s modified Eagle’s medium (DMEM) with L-glutamine and sodium pyruvate, fetal bovine serum (FBS), 0.25% Trypsin/0.02% EDTA solution and Penicillin/Streptomycin 100x solution (Pen/Strep) were purchased from Biowest (Nuaillé, France). Phosphate buffer saline tablets (PBS) were from Sigma Aldrich (St Louis, MO, USA). Dimethyl sulfoxide (DMSO) (CAS 67–68-5, >99.9%) and 3-[4,5-dimethylthiazol-2-yl]-2,5-diphenyltetrazolium bromide (MTT) (CAS 298–93-1, >98.0%) was purchased from Duchefa Biochemie B.V. (Haarlem, Netherlands).

### 2.2 Cell culture

Human neuroblastoma SH-SY5Y cell line (ACC 209, LOT 17) was purchased from DSMZ (Leibniz Institute, GmbH). SH-SY5Y were routinely cultured in 75 cm^2^ culture flasks (Sarstedt; Nümbrecht, Germany) in complete cell culture medium (CM) composed of DMEM, supplemented with 15% FBS (v/v) and 1% Pen/Strep (v/v) in a humidified incubator at 37 °C and 5% CO_2_. Subculture procedures were performed once a week at 80%–90% cell confluence. Following CM removal, adherent cells were rinsed with pre-warmed sterile PBS and detached using 1 mL of 0.25% trypsin–EDTA for 2 min at 37 °C. Experiments were conducted with undifferentiated SH-SY5Y cells at passage numbers below 15 to ensure the preservation of their neuronal profile (Prisacar et al., 2025).

### 2.3 Preparation of contaminants stock solutions

Stock solutions of AFB1 (35 mM) were prepared in DMSO, while ACR (10 M) and Cd^2+^ (1 M) were prepared in ultrapure water. Stock solutions were stored at −20 °C and filtered-sterilized working solutions were prepared in CM right before experiments. The final DMSO concentration in treatment conditions did not exceed 0.5% (v/v).

### 2.4 Cell viability assay

Cell viability was assessed using the MTT assay after exposure to individual contaminants and binary and ternary mixtures. The assay measures the activity of intracellular NAD(P)H-dependent oxidoreductases, which reduce the yellow tetrazolium salt MTT to insoluble purple formazan crystals, in proportion to the number of metabolically active cells (Ghasemi et al., 2021). Cell-free control experiments confirmed the absence of direct interference of the tested compounds with the MTT reduction reaction. SH-SY5Y cells were seeded in 96-well plates at 10,000 cells/well and allowed to adhere for 24 h at 37 °C and 5% CO_2_ before exposure. Cells were then exposed to test compounds for 72 h. After exposure, the culture medium was removed and 100 µL of MTT solution (0.5 mg/mL) was incubated for 3 h. Formazan crystals were then dissolved in 100 µL DMSO, and absorbance was recorded at 570 nm using a SPECTROstar Nano microplate reader (Ortenberg, Germany). Data were obtained from at least three independent experiments with six technical replicates per concentration. Results are expressed as percentage cell viability relative to adequate solvent control.

For the individual contaminants, concentration-response curves AFB1, ACR, and Cd^2+^ were generated using serial 2-fold dilutions. AFB1 and Cd^2+^ were tested from 0.78 to 100 μM, whereas ACR was tested over a range of 78.13–10,000 µM. Binary and ternary mixtures were prepared using fixed equipotent ratios derived from the IC_50_ values obtained by CT modeling of the single-compound concentration-response curves. Based on these IC_50_ estimates, binary mixtures were designed at ACR/AFB1 = 80:1, ACR/Cd^2+^ = 160:1, and AFB1/Cd^2+^ = 2:1, while the ternary mixture was prepared at a fixed ratio of ACR/AFB1/Cd^2+^ = 160:2:1. This strategy standardizes the contribution of each component at the median-effect level, but the equivalence is formally valid only at IC50. Because relative potency can shift across the response range when slope values differ, departures from equipotency at lower or higher effect levels were considered an expected limitation of the fixed-ratio design.

### 2.5 Calculation of predicted mixture effects using different reference models

The identification of synergy, antagonism, or additivity in mixtures requires an accurate estimation of the expected joint effect.

#### 2.5.1 Independent action

The IA model, also known as Bliss independence, assumes that each compound acts independently. The expected joint effect is the probabilistic combination of the individual effects. For a binary mixture, the predicted fraction affected (fa) is:

$${Fa}_{AB}={Fa}_{A}+{Fa}_{B}-{Fa}_{A}\times {Fa}_{B}$$

Where Fa_AB_ is the expected fraction of cells affected (non-viable) by the mixture at the specific concentration tested, and Fa_A_ and Fa_B_ correspond to the fractions of non-viable cells induced by compounds A and B individually at the same concentrations used in the mixture.

#### 2.5.2 Chou-Talalay method

The Chou-Talalay method is one of the most widely used methods to analyze the interactions between compounds (Chou, 2006). The basis of this method is the median-effect equation of the mass-action law, which unifies the Michaelis-Menten equation for enzyme substrate saturation, the Hill equation for ligand occupancy at high order, the Henderson-Hasselbalch equation for pH ionization, and the Scatchard equation for the receptor binding:

$$\frac{Fa}{Fu}={\left(\frac{D}{D_{m}}\right)}^{m}$$

where *fa* is the fraction of cells affected (non-viable), *fu* is the fraction of cells unaffected (i.e., viable) and corresponds to 1-fa, D_m_ is the dose that produces 50% of the maximum effect (i.e., the IC_50_), and *m* describes the shape of the dose-response.

To estimate these parameters, experimental data are fitted to the linearized form of the median-effect equation (MEE):

$$log\left(\frac{Fa}{Fu}\right)=m\times \log\left(D\right)-m\times \log\left(D_{m}\right)$$

#### 2.5.3 CISNE method

CISNE (Code for the Identification of Synergism Numerically Efficient) was developed as an improvement to the CT framework. Instead of linearizing the MEE, CISNE fits the experimental points directly to the nonlinear median-effect function using nonlinear least-squares algorithms. This approach offers two key advantages. First, all data points receive the same statistical weight, independent of the value of *fa*, reducing bias introduced by logarithmic transformation. Second, data points with *fa* ≥ 1 or *fa* ≤ 0 are retained rather than discarded, which is particularly relevant when modelling FC exposures where concentrations relevant for human exposure produce minimal effects (*fa* ≤ 0).

### 2.6 Quantification of interactions between food contaminants under multiple reference models

Interactions between compounds were evaluated using combination index (CI) values.

For CT and CISNE, CI values were calculated as established by Chou and Talalay (Chou, 2006):

$${CI}_{x}=\frac{D_{1}}{{D_{x}}_{1}}+\frac{D_{2}}{{D_{x}}_{2}}$$

where CI_x_ is the combination index at *x*% effect, *D*_*1*_ and *D*_*2*_ are the doses of each compound in the mixture that produce *x*% effect, and *D*_*x*_ *,*_*1*_ and *D*_*x*_ *,*_*2*_ are the corresponding doses required for each compound alone to achieve the same effect level. For interpretation, CI thresholds of CI < 0.9, 0.9 ≤ CI ≤ 1.1, and CI > 1.1 were used to indicate synergism, additivity and antagonism, respectively (Chou, 2006).

In addition, the dose-reduction index (DRI), was determined to estimate the fold reduction in the dose of each contaminant in combination relative to the dose required when administered alone to achieve the same effect level (x%). For a binary mixture, DRI values were calculated for each compound as:

$$DRI_{1}=\frac{{\left(D_{x}\right)}_{1}}{D_{1}},DRI_{2}=\frac{{\left(D_{x}\right)}_{2}}{D_{2}}$$

where ${\left(D_{x}\right)}_{1}$ and ${\left(D_{x}\right)}_{2}$ represent the doses of each contaminant applied individually to produce an effect of x%, and $D_{1}$ and $D_{2}$ correspond to the doses of each contaminant within the binary mixture that produced the same effect. A DRI equal to 1 indicates no dose reduction in combination, whereas DRI values greater than 1 indicate that a lower dose is required in the mixture to achieve the specified effect level, relevant in the context of synergistic interactions.

### 2.7 Data and statistical analysis

Data were expressed as mean percentage cell viability ± standard error of the mean (SEM) and plotted using GraphPad Prism 9.3.1 for Windows (GraphPad Software, La Jolla, CA, USA). Experimental datasets were screened for outliers using the Grubbs test. Outlier exclusion was limited to isolated technical replicates within individual experimental runs. No concentration or treatment group was excluded in full, and removal of these values did not alter the fitted concentration–response trends or the qualitative interaction classification. Statistical comparisons with the corresponding control group were performed using one-way ANOVA followed by Dunnett’s *post hoc* test.

For concentration–effect curve analysis, viability data from each independent assay were converted to cell death (1 − x), corresponding to the Fa. For the independent action (IA) model, concentration–effect data for each individual contaminant were fitted to non-linear functions derived from the Hill equation, using fixed or variable slope models. As a requirement of IA, equations were constrained to bottom = 0 and top = 1. Accordingly response values were constrained to the 0–1 interval, with 0.0001 corresponding to the lowest value for the respective condition or blank and 0.9999 to the highest value or control. This was required for AFB1, ACR/Cd^2+^ and ACR/AFB1/Cd^2+^. Model selection was based on the lowest Akaike information criterion (AIC), and the selected model was used to estimate IC_x_ values and generate IA-based mixture predictions. For the Chou–Talalay (CT) approach, concentration–effect data were fitted to the linearized form of the median-effect equation (MEE) using CompuSyn version 1.0 (ComboSyn Inc., Paramus, NJ, USA). Because CT requires 0 < Fa < 1, the same normalization and constraint procedures applied for IA were used. CT fitting was performed using both mean Fa values per concentration and replicate-level Fa values to evaluate the impact of data aggregation on parameter estimation. Goodness-of-fit was assessed using the linear correlation coefficient (r) of the median-effect plot, and R^2^ values were additionally calculated from the corresponding non-linear MEE using the derived parameters. CISNE implementation was applied by directly fitting the non-linear MEE using CISNE version 1.0 (University of Oviedo). In this approach, Fa values from each independent assay were entered after normalization to their respective controls, without imposing lower or upper constraints. Parameter estimates (Dm and m) and their associated uncertainty were obtained directly from the non-linear regression. IC_50_ estimates obtained from CT fitting were used to define fixed equipotent mixture ratios. Mixture concentration–effect data were analysed using the same modelling frameworks as for single compounds, and CI values were computed with corresponding 95% confidence intervals.

## 3 Results

### 3.1 Cytotoxicity of single chemical contaminants

**FIGURE 1:**
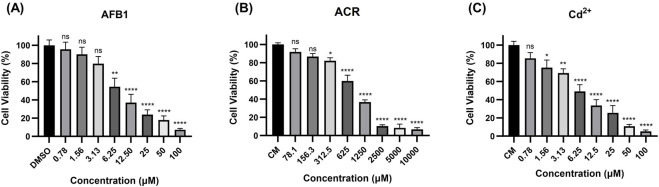
Effects of **(A)** aflatoxin B1, **(B)** acrylamide, and **(C)** Cd^2+^ on human neuroblastoma SH-SY5Y cells viability following 72 h exposure. Data represent percentage cell viability relative to the respective control (mean ± SEM), using complete medium (CM) as the control for acrylamide and cadmium, and 0.5% DMSO as the solvent control for aflatoxin B1. For aflatoxin B1, no differences were observed between medium and solvent controls, only the solvent control is shown. Sample sizes were N = 3 for acrylamide and aflatoxin B1, and N = 4 for cadmium. Statistical differences relative to control were assessed by one-way ANOVA with Dunnett’s post-hoc test. Significance symbols: ns, not significant; * p < 0.05; ** p < 0.01; *** p < 0.001; **** p < 0.0001 versus control.

All contaminants decreased cell viability in a concentration-dependent manner, with overall potency ranked Cd^2+^ > AFB1 > ACR (Figure 1). Cd^2+^ induced a significant reduction in viability at 1.56 µM (75.18% viability, p < 0.05 vs. CM), followed by a steep concentration-dependent decline, with viability reduced to below 10% at ≥ 50 µM (p < 0.0001). AFB1 caused significant cytotoxicity from 6.25 µM onwards (approximately 54.5% viability, p < 0.01 vs. DMSO), with viability dropping below 40% at 12.5 µM and approaching minimal levels at 50–100 µM (p < 0.0001). In contrast, ACR showed the lowest potency, with the first significant decrease detected at 312.5 µM (82.1% viability, p < 0.05 vs. CM) with marked cytotoxicity observed at high micromolar to millimolar concentrations (p < 0.0001).

Experimental cytotoxicity data for each contaminant were then fitted to the Hill function, and the resulting parameter estimates are summarized in Table 1. Full curve fits are provided in Supplementary Figure S1. Notably, the Hill slope differed considerably among FCs, indicating differences in concentration-response curve shape and relative potency across effect levels, which influence the suitability of concentration addition-based assumptions. This aspect is further discussed in Section 4.4. Data were also fitted to the MEE using two approaches: within the CT framework, parameters were estimated from the linearized MEE, whereas CISNE applied direct non-linear regression of the MEE to the experimental data (Table 1). To meet the assumptions required for the independent action (IA) and CT models, the experimental data was constrained between 0 and 1 as described in Section 2.7 for both Hill and CT models. Comparison between the linearized CT and direct non-linear CISNE implementations revealed relatively similar IC50 estimates for the individual compounds, although larger deviations emerged at lower and higher effect levels. CT-derived values were used for mixture design to maintain consistency with the classical CT equipotent ratio approach.

**TABLE 1:** Estimation of concentration–response parameters and inhibitory concentrations (IC_10_–IC_90_) for aflatoxin B1 (AFB1), acrylamide (ACR), and Cd^2+^ in SH-SY5Y cells after 72 h exposure. Experimental cytotoxicity data were fitted to the Hill function and to the median-effect equation (MEE) using two implementations: the linearized approach within the Chou–Talalay (CT) framework and the direct non-linear regression implemented in CISNE. To satisfy the assumptions of the independent action (IA) and CT models, replicate data were constrained between 0 and 1 (0 corresponding to the minimum response and 1 to the maximum response within each independent experiment). Reported parameters include the slope (Hill slope or MEE parameter m) and goodness-of-fit (R^2^). Values in parentheses denote 95% profile-likelihood confidence intervals.

Compound	Model^a^	Slope	R²	IC_10_	IC_25_	IC_50_ (Dm)	IC_75_	IC_90_
AFB1 (0.8–100 µM)	Hill	1.06 (0.81–1.33)	0.93	1.01	2.83	7.96 (6.45–9.88)	22.39	62.92
	Chou–Talalay	1.33 (0.94-1.71)	0.70	2.01	4.62	10.58	24.25	55.56
	CISNE	1.13 (0.86-1.50)	0.90	1.24	3.28	8.71 (6.74–11.31)	23.13	61.42
ACR (78.1–10000 µM)	Hill/CISNE	1.41 (1.18–1.71)	0.97	168.5	367.9	803.2 (700.6–919.7)	1753.6	3826.6
	Chou–Talalay	1.24 (1.09-1.40)	0.92	128.3	311.9	758.4	1843.9	4483.1
Cd²⁺ (0.8–100 µM)	Hill/CISNE	0.91 (0.72–1.14)	0.87	0.55	1.85	6.24 (4.84–8.00)	20.99	70.58
	Chou–Talalay	1.08 (0.88-1.27)	0.81	0.85	2.37	6.57	18.24	50.65

^a^ CISNE implementation of the MEE is mathematically equivalent to a two-parameter Hill (logit) model; therefore, when response data are constrained between 0 and 1, the estimated parameters are identical between both approaches.

### 3.2 Cytotoxicity of binary and ternary mixtures of chemical contaminants

**FIGURE 2:**
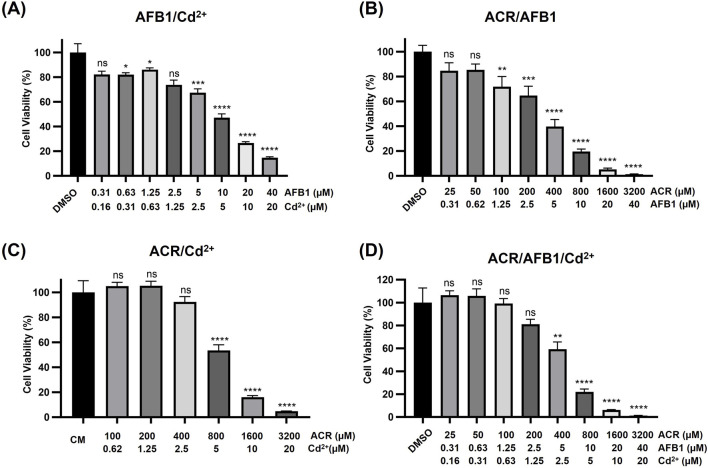
Effects on human neuroblastoma SH-SY5Y cells viability of binary mixtures **(A)** AFB1/Cd^2+^, **(B)** ACR/AFB1, **(C)** ACR/Cd^2+^, and the ternary mixture **(D)** ACR/AFB1/Cd^2+^ following 72 h exposure. Cell viability was assessed by the MTT assay and is expressed as percentage of the respective control mean ± SEM, using CM as the control for mixtures containing ACR and/or Cd^2+^ and 0.5% DMSO as the solvent control for mixtures containing AFB1. Sample sizes were N = 3 for binary mixture, and N = 4 for ternary mixture. Statistical differences relative to the corresponding control were determined by one-way ANOVA followed by Dunnett’s multiple comparisons test. Significance symbols: ns, not significant; * p < 0.05; ** p < 0.01; *** p < 0.001; **** p < 0.0001 versus control.

Binary and ternary mixtures were subsequently prepared using serial dilutions at a fixed relative ratio, based on individual IC_50_ values derived from CT fits (Table 1): 2:1 for AFB1/Cd^2+^, 80:1 for ACR/AFB1, 160:1 for ACR/Cd^2+^, and 160:2:1 for ACR/AFB1/Cd^2+^. The concentration ranges tested are shown in Figure 2 and Table 2.

**TABLE 2:** Hill function fits for binary and ternary mixtures of aflatoxin B1 (AFB1), acrylamide (ACR) and Cd^2+^ in SH-SY5Y cells after 72 h exposure (MTT assay). For each mixture, the tested dose range and mixture ratio are reported together with the fitted Hill slope, goodness-of-fit (R^2^), and the half-maximal inhibitory concentration (IC_50_). To satisfy the assumptions of the independent action (IA) model, replicate data were constrained between 0 and 1 (0 corresponding to the minimum response and 1 to the maximum response within each independent experiment). Values in parentheses denote 95% profile-likelihood confidence intervals.

Mixture	Dose Range	Ratio	Hill Slope	R_2_	IC_50_
AFB1/Cd^2+^	AFB1: 0.31 – 40 µM Cd^2+^: 0.16 – 20 µM	2:1	0.86 (0.79-1.1)	0.92	11.79 (9.60-14.50)
ACR/AFB1	AFB1: 0.31 – 40 µM ACR: 25 – 3200 µM	80:1	1.15 (0.92-1.47)	0.93	261.2 (209.3-324.1)
ACR/Cd^2+^	ACR: 25 – 3200 µM Cd^2+^: 0.16 – 20 µM	160:1	2.59 (2.37-2.84)	1.00	817.3 (786.7-849.3)
ACR/AFB1/Cd^2+^	ACR: 25 – 3200 µM AFB1: 0.31 – 40 µM Cd^2+^: 0.16 – 20 µM	160:2:1	1.72 (1.53-1.95)	0.98	394.0 (363.5-426.8)

All mixtures decreased SH-SY5Y viability in a concentration-dependent manner. Based on the fitted IC_50_ values in Table 2, mixture potency ranks AFB1/Cd^2+^ (11.79 µM) > ACR/AFB1 (261.2 µM) > ACR/AFB1/Cd^2+^ (394.0 µM) > ACR/Cd^2+^ (817.3 µM). AFB1/Cd^2+^ mixture exhibited the lowest IC_50_ value, reflecting the relatively high individual potencies of both components, whereas ACR/Cd^2+^ showed the lowest mixture potency, consistent with the weaker cytotoxicity of ACR as a single compound. To provide an initial qualitative baseline of joint toxicity, the experimental IC_50_ values of the binary and ternary mixtures were evaluated using a response additivity framework. For ACR/AFB1 mixture, the observed effect exceeded the predicted arithmetic sum of the individual component effects (E_predicted_ = 0.44), suggesting a slight deviation towards synergism. In contrast, all other mixtures showed lower-than-expected effects: AFB1/Cd^2+^ (E_predicted_ = 0.89), ACR/Cd^2+^ (E_predicted_ = 0.96), and the ternary mixture (E_predicted_ = 0.93), consistent with antagonistic behavior under this metric.

### 3.3 Evaluation of contaminants interactions in binary and ternary mixtures

#### 3.3.1 Comparison of observed mixture responses with predicted response from independent action model

**FIGURE 3:**
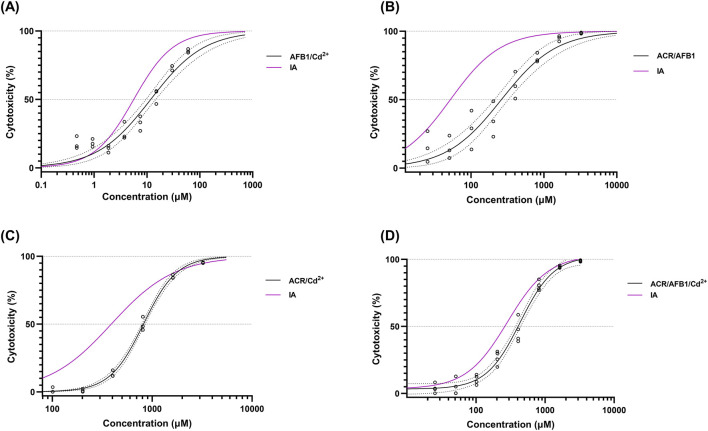
Comparison of experimentally observed cytotoxicity with independent action (IA) model predictions for fixed equipotent ratio mixtures in SH-SY5Y cells following 72 h exposure (MTT assay): **(A)** AFB1/Cd^2+^ (2:1), **(B)** ACR/AFB1 (80:1), **(C)** ACR/Cd^2+^ (160:1), and **(D)** ACR/AFB1/Cd^2+^ (160:2:1). Symbols represent experimental data points. Solid black lines correspond to the Hill fitted concentration–response curves for the observed mixture data, with dotted black lines indicating the 95% confidence intervals of the fit. The colored line represents IA-predicted effects. Horizontal dashed lines indicate 50% and 100% cytotoxicity.

A comparison of the experimentally observed concentration–response curves with IA-predicted responses is presented in Figure 3. If the experimentally observed effect is greater than the IA-predicted effect at a given concentration, the mixture is interpreted as synergistic; if it is lower, the interaction is interpreted as antagonistic.

For the AFB1/Cd^2+^ combination (Figure 3A), the IA-predicted curve is left-shifted relative to the experimental response, indicating that IA overestimates mixture toxicity across most of the concentration range. This pattern reflects overall antagonistic behavior in relation to IA curve estimation. However, at the lowest concentrations tested, the experimental and IA curves largely overlap, supporting additive behavior in the low-dose region. For the ACR/AFB1 mixture (Figure 3B), the observed concentration–response curve closely followed IA predictions across the concentration range, indicating predominantly additive behavior relative to IA. For the ACR/Cd^2+^ mixture (Figure 3C), experimentally observed cytotoxicity was consistently lower than IA-predicted values at low and intermediate concentrations, indicating antagonistic interaction relative to IA. The divergence between curves was most evident at low fractional effects, suggesting that antagonism is more pronounced in the low-effect range. Similarly, for the ternary ACR/AFB1/Cd^2+^ mixture (Figure 3D), the experimentally observed cytotoxicity was generally lower than the IA-predicted values throughout most of the curve, indicating an antagonistic interaction.

#### 3.3.2 Chou–Talalay and CISNE-based interaction analysis

To compare the impact of different MEE implementations on the assessment of contaminant interactions in mixtures, experimental mixture cytotoxicity data were fitted using both the classical CT method and the non-linear implementation in CISNE (Table 3).

**TABLE 3:** Median-effect equation (MEE) parameter estimates and derived effect concentrations for fixed equipotent ratio mixtures in SH-SY5Y cells following 72 h exposure. Experimental cytotoxicity data were fitted to the MEE using two approaches: the linearized implementation within the Chou–Talalay (CT) framework and direct non-linear regression as implemented in CISNE. To meet CT model requirements, data were constrained between 0 and 1 (0 corresponding to the minimum response and 1 to the maximum response within each independent experiment). Reported parameters include the slope (MEE parameter, m) and goodness-of-fit (R^2^). Values in parentheses indicate 95% profile-likelihood confidence intervals.

Mixture (ratio)	Model	Slope	R²	IC_10_	IC_25_	IC_50_ (Dm)	IC_75_	IC_90_
AFB1/Cd^2+^ (2:1)	CISNE	0.86 (0.70-1.06)	0.92	0.91	3.28	11.79	42.32	151.9
	Chou–Talalay	0.72 (0.59‐0.85)	0.85	0.49	2.24	10.25	46.97	215.6
ACR/AFB1 (80:1)	CISNE	1.15 (0.92-1.47)	0.93	38.78	100.6	261.2	678.0	1759.7
	Chou–Talalay	1.32 (1.12-1.51)	0.90	37.95	87.50	201.8	465.2	1072.7
ACR/Cd^2+^ (160:1)	CISNE	2.93 (2.35-3.85)	0.98	412.1	598.9	870.3	1264.9	1838.3
	Chou–Talalay	2.94 (2.37-3.50)	0.88	451.9	656.6	953.9	1385.9	2013.5
ACR/AFB1/Cd^2+^ (160:2:1)	CISNE	2.17 (1.77-2.73)	0.96	168.6	280.0	464.9	772.1	1281.1
	Chou–Talalay	1.99 (1.63-2.35)	0.81	126.7	220.0	382.1	663.5	1152.1

Overall, the CISNE non-linear fitting consistently provided higher goodness-of-fit (R^2^ = 0.92–0.98) compared with the CT linearized approach (R^2^ = 0.89–0.97), indicating improved model performance when fitting the full, untransformed dose–response data. Slope (m) estimates obtained with CT were generally higher, particularly for ACR/Cd^2+^ and the ternary mixture, suggesting steeper concentration–response relationships under the linearized framework. This translated into systematically higher IC values under CT for some mixtures, most notably ACR/Cd^2+^, where CT predicted a right-shifted response relative to CISNE, whereas for ACR/AFB1, CT yielded lower IC_50_ values, indicating a left-shift. The practical impact of model choice is evident in the AFB1/Cd^2+^ mixture estimates. While IC_50_ values showed only a modest divergence (10.25 μM for CT vs. 11.79 μM for CISNE; ∼13%), differences became more pronounced at the extremities of the concentration-response curve. At the IC_10_ level, the CT model estimated 0.49 μM, corresponding to a 46% difference relative to the CISNE estimate of 0.91 μM. Conversely, at the IC_90_ level, the CT estimate (215.6 μM) was 42% higher than CISNE (151.9 μM).

Data pre-processing represents a critical step for reliable interaction estimation. Comparison of data input strategies showed that fitting based on mean responses yielded, as expected, higher goodness-of-fit than fitting individual replicate data in both the CT and CISNE frameworks (Supplementary Table S1). When comparing both models in fitting replicate datasets under constrained conditions, the CISNE framework provided more stable parameter estimation than the CT approach (Supplementary Table S1). These results highlight that although averaging improves apparent goodness-of-fit, direct fitting of replicate data better captures experimental variability and may provide a more statistically robust basis for mixture interaction analysis.

#### 3.3.3 Combination index as a metric of quantification of interactions

**FIGURE 4:**
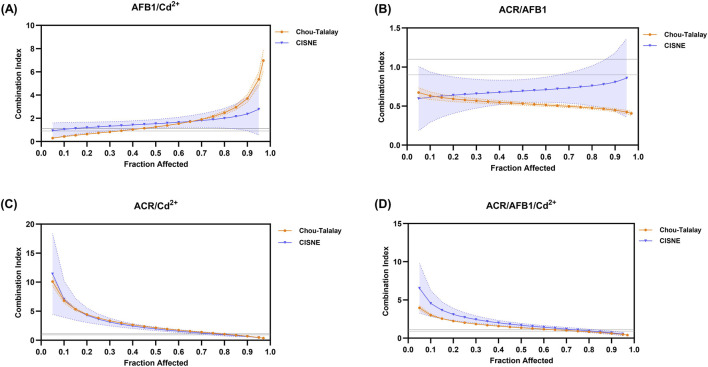
Fa-CI plots for **(A)** AFB1/Cd^2+^ (2:1), **(B)** ACR/AFB1 (80:1), **(C)** ACR/Cd^2+^ (160:1), and **(D)** ACR/AFB1/Cd^2+^ (160:2:1). CI values were calculated from Chou-Talalay model (orange) and CISNE (blue). Shaded areas that accompany the plots represent the 95% confidence intervals for CI values calculated in Compusyn and CISNE software, respectively. Horizontal dashed gray lines delimit the additive zone (0.9 ≤ CI ≤ 1.1), separating synergistic interactions (CI < 0.9) from antagonistic interactions (CI > 1.1).

CI values were calculated for all binary and ternary mixtures across a wide range of Fa values using combination index approaches based on the CT and CISNE models (Supplementary Table S2) and are visualized in Figure 4. In addition to Fa–CI plots, the dose reduction index (DRI) was determined to estimate the fold reduction in the concentration of each compound in the mixture required to achieve a defined effect level relative to single-compound exposure (Table 4).

**TABLE 4:** Dose reduction index values (DRI) pertaining to (A) Chou-Talalay model and (B) CISNE model at inhibition concentration of 10, 25, 50, 75% and 90% for AFB1/Cd^2+^, ACR/AFB1, ACR/Cd^2+^, and ACR/AFB1/Cd^2+^ mixtures. DRI values are highlighted in a white-green gradient (from 0 to maximum DRI found, respectively) for easier visualization.

(A) Chou-Talalay							
Mixture	Ratio	Compound	IC10	IC25	IC50	IC75	IC90
AFB1/Cd^2+^	2:1	AFB1	6.19	3.10	1.55	0.77	0.39
		Cd^2+^	5.24	3.18	1.92	1.17	0.71
ACR/AFB1	80:1	ACR	3.42	3.61	3.81	4.01	4.23
		AFB1	4.30	4.27	4.25	4.22	4.20
ACR/Cd^2+^	160:1	ACR	0.29	0.48	0.80	1.34	2.24
		Cd^2+^	0.30	0.58	1.11	2.12	4.05
ACR/AFB1/Cd^2+^	160:2:1	ACR	1.03	1.44	2.02	2.83	3.96
		AFB1	1.30	1.71	2.26	2.98	3.93
		Cd^2+^	1.10	1.75	2.80	4.48	7.17
(B) CISNE model							
AFB1/Cd^2+^	2:1	AFB1	2.03	1.50	1.11	0.82	0.61
		Cd^2+^	1.81	1.69	1.59	1.49	1.39
ACR/AFB1	80:1	ACR	4.40	3.70	3.11	2.62	2.20
		AFB1	2.58	2.64	2.70	2.76	2.83
ACR/Cd^2+^	160:1	ACR	0.41	0.62	0.93	1.39	2.09
		Cd^2+^	0.22	0.50	1.15	2.67	6.18
ACR/AFB1/Cd^2+^	160:2:1	ACR	1.02	1.34	1.76	2.31	3.04
		AFB1	0.60	0.96	1.53	2.44	3.90
		Cd^2+^	0.53	1.08	2.19	4.43	8.97

Relevant divergences appear for the AFB1/Cd^2+^ mixture (Figure 4A), with model-dependent classification at low effect levels. At Fa 0.05, CT indicates clear synergism (CI 0.30), while CISNE estimates a nearly additive interaction (CI 0.93) (Supplementary Table S2). At Fa 0.10, CT classifies the mixture as synergistic (CI 0.43), whereas CISNE shifts toward slight antagonism (CI 1.05). As the Fa increases, both models predict antagonistic interactions between AFB1 and Cd^2+^. For the ACR/AFB1 mixture (Figure 4B), the CT model indicated synergism across the entire Fa range, with CI values decreasing from 0.67 at Fa 0.05 to 0.45 at Fa 0.9, reflecting stronger synergism at higher effect levels (Supplementary Table S2). CISNE also classified the interaction as synergistic, ranging from slight to moderate. At low Fa levels, CISNE predicted stronger synergism than CT, with CI values 0.61 at Fa 0.10 (Supplementary Table S2). This interaction translated into marked dose reductions for both compounds, with CISNE indicating that, within the mixture, approximately 4.4-fold less ACR and 2.6-fold less AFB1 were required to achieve the same effect compared with individual exposures (Table 4). The ACR/Cd^2+^ combination (Figure 4C) exhibited pronounced antagonism at low Fa values in both models, with CI values progressively approaching the additive range as Fa increased. CT calculated higher CI values across Fa levels compared to CISNE model. The ternary mixture ACR/AFB1/Cd^2+^ (Figure 4D) both models depict antagonistic tendencies at low-to-intermediate Fa values, with CI values gradually decreasing toward additivity at higher effect levels. At low Fa values, both ACR/Cd^2+^ and ternary mixture exhibit DRI below 1, indicate that a higher dose is required in the mixture than in single-compound exposure to achieve the same effect level (i.e., DRI 0.22 for Cd^2+^ in mixture with ACR).

**FIGURE 5:**
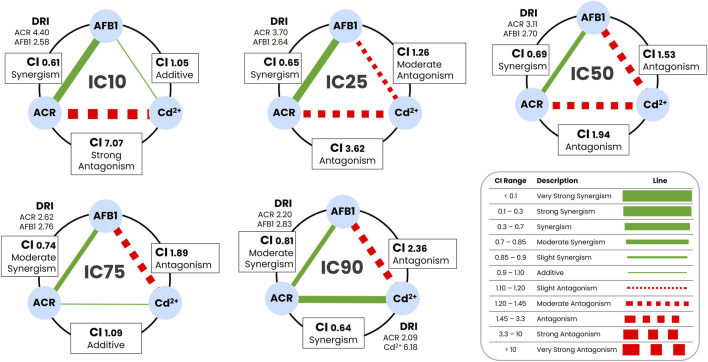
Polygonograms representing all binary combinations interactions at IC_10_, IC_25_, IC_50_, IC_75_ and IC_90_. Edges represent pairwise interactions and are labeled with the corresponding combination index (CI) values derived from the CISNE implementation of the median-effect equation. Dose reduction index (DRI) values are indicated where synergistic interactions were observed. Line symbology represent interactions magnitude corresponding to different CI intervals.

Figure 5 presents the polygonograms for all binary mixtures at five inhibition levels (IC_10_, IC_25_, IC_50_, IC_75_ and IC_90_), allowing comparison of interaction trends across increasing effect magnitudes. These were derived from the CISNE method, which provided consistently a superior fit to the experimental data, as indicated by goodness-of-fit metrics (Table 3), thereby offering a more reliable basis for quantitative interaction assessment.

## 4 Discussion

### 4.1 Cytotoxicity of individually exposed contaminants to the SH-SY5Y neuronal cells

The SH-SY5Y cell line is commonly used to evaluate the impact of chemical pollutants on the nervous system (Lopez-Suarez et al., 2022). According to Lopez-Suarez et al. (2022), undifferentiated SH-SY5Y cells are the predominant model in the field, featured in at least 80% of 163 reviewed neurotoxicity studies. While most existing research focuses on short-term exposure - typically 24 h and occasionally 48 h - relatively few studies extend to 72 h. To address this gap, this study exposed undifferentiated SH-SY5Y cells to FCs for a 72-h period. This extended duration was chosen to allow for comparison with existing data while better capturing the cumulative and delayed cytotoxicity responses, which are more consistent with the sustained cellular stress underlying neurodegenerative processes.

AFB1 is easily absorbed through the gastrointestinal tract (Song et al., 2024), and has been detected in the brain of mice following oral exposure (Huang et al., 2020; Lin et al., 2025a). In humans, AFB1 and its metabolites exhibited biphasic kinetics, characterized by an initial rapid plasma elimination phase with a half-life of approximately 2.86 h, followed by a slower terminal phase with a half-life of 64.4 h (Jubert et al., 2009). In rats exposed orally to 8.12 μg/kg of AFB1, blood concentration peaked at 4/5 h post-administration, with a half-life of approximately 64 h (Song et al., 2024). Moreover, AFB1 can also form covalent adducts with lysine residues in albumin, resulting in AFB1–lys complexes (Arce-Lopez et al., 2020). Given that albumin has a circulating half-life of about 20 days, these adduct levels are considered biomarkers of cumulative exposure over the preceding 6–8 weeks [EFSA Panel on Contaminants in the Food Chain (CONTAM) et al., 2020].

In the present study, AFB1 exhibited neurotoxicity in SH-SY5Y cells, with an IC_50_ of 7.97 µM (Table 1). This value is consistent with previous reports in human neuroblastoma models, including IMR-32 cells, where an IC_50_ of 6.18 μg/mL (19.8 µM) was reported after 24 h exposure (Huang et al., 2020), and SK-N-SH cells, where AFB1 decreased cell viability to 58% after incubation of 10 μg/mL (32 µM) for 48 h (Bonsi et al., 1996). Importantly, *in vivo* evidence supports the neurodegenerative potential of AFB1. Oral administration of AFB1 (15.75 μg/kg for 8 weeks; equivalent to 0.50 µM) in Wistar rats induced marked astrocytic loss in the frontal cortex and neuronal degeneration in the hippocampus (Bahey, Abd Elaziz, and Gadalla, 2015). These histopathological alterations were partially reversible following cessation of exposure (Bahey, Abd Elaziz, and Gadalla, 2015). In the same strain, exposure to 25 μg/kg AFB1 produced time-dependent neurodegenerative effects, with detectable neuronal damage after 30 days and more pronounced after 90 days, consistent with cumulative toxicity (Alsayyah et al., 2019). In addition to oxidative stress, these studies reported vascular alterations, including endothelial relaxation, vasodilation, and impairment of blood–brain barrier integrity (Bahey et al., 2015; Alsayyah et al., 2019). Similarly, in C57BL/6J mice, sustained oral exposure to AFB1 at approximately 0.18 mg/kg/day (around 0.58 µM) for 8 weeks led to cognitive decline, hippocampal neuronal degeneration, disruption of blood-brain barrier integrity, and increased expression of AD-associated markers, including App, Bace1, Psen1, and phosphorylated Tau (Lin et al., 2025a; Lin et al., 2025b). Mechanistically, AFB1 accumulated within hippocampal tissue, disrupted redox homeostasis, and activated ferroptotic pathways, supporting iron-dependent lipid peroxidation as a key driver of AFB1-induced neurodegeneration (Lin et al., 2025a; Lin et al., 2025b). Although the *in vivo* exposure levels reported in animal studies are substantially lower than the *in vitro* IC50 values observed here, direct quantitative comparisons remain limited by differences in exposure duration, toxicokinetics, bioaccumulation, metabolism, and the simplified nature of *in vitro* systems, which often require higher nominal concentrations to elicit measurable acute effects. Nevertheless, these findings extend the toxicological profile of AFB1 beyond its established carcinogenicity and position this mycotoxin as a plausible contributor to NDs development under sustained exposure conditions.

ACR is a well-established neurotoxicant, historically associated with peripheral neuropathy following occupational exposure (EFSA CONTAM Panel, 2015; Rifai and Saleh, 2020). For non-smokers, diet represents the primary route of exposure, with ACR formed during high-temperature processing of carbohydrate-rich foods via Maillard reactions involving reducing sugars and asparagine (Mottram et al., 2002). Increasing attention has focused on early-life exposure due to its ability to cross the placental barrier and its detection in breast milk (Lindeman et al., 2021). Experimental studies indicate that chronic low-dose ACR exposure induced neuronal loss, altered neurotransmitter systems, and led to persistent behavioral impairments in rodents (EFSA CONTAM Panel, 2015; Rifai and Saleh, 2020), while epidemiological data suggest associations between dietary ACR intake and cognitive decline in older adults (Z.M. Liu et al., 2017). Consistent with previous *in vitro* studies, ACR required higher concentrations to induce cell death in SH-SY5Y neuronal cells, with an IC_50_ of 803.2 µM (Table 1). Attoff et al., reported an IC_50_ of 1,000 µM following 72 h exposure using the MTT assay (Attoff et al., 2016). Bridgeman et al. reported progressive increases in ACR-induced toxicity between 24, 48 and 72 h in SH-SY5Y cells, confirming that longer incubations amplify the potency of this contaminant (Bridgeman et al., 2023).

Cadmium places 7^th^ on the Priority List of the Agency for Toxic Substances and Disease Registry based on their frequency, toxicity, and potential for human exposure (Registry, Agency for Toxic Substances and Disease, 2024). It is a widespread environmental contaminant, with a biological half-life of up to 20 years, resulting in the progressive bioaccumulation in soft tissues, primarily the liver and kidneys (Jomova et al., 2024). Beyond systemic accumulation, Cd represents a major concern in the context of neurotoxicity.

In the present study, Cd^2+^ was the most cytotoxic compound tested, with an IC_50_ of 6.24 µM after 72 h exposure (Table 1). Mallamaci et al. (2024) reported an IC_50_ of 2.5 µM under similar experimental conditions, highlighting consistent vulnerability of human neuroblastoma cells to prolonged Cd^2+^ exposure. In contrast, shorter exposures (24 h) in SH-SY5Y cells have yielded higher IC_50_ values, such as 67.5 µM for ATP depletion, reflecting lower sensitivity compared to other neuronal lines as N1E-115 and PC12 (Mundy et al., 2010). Similarly, Bovio et al. observed only a partial viability reduction (63%) at 20 µM after 24 h using the MTT assay (Bovio et al., 2021). Together, these findings underscore the importance of longer exposure durations in capturing the cumulative cytotoxic potential of heavy metals.

Additionally, studies have suggested that overall heavy metal exposure may contribute to the development or progression of NDs through several potential mechanisms such as oxidative stress, calcium imbalance, neuroinflammation (Nisa et al., 2021). Chronic Cd exposure can lead to disruption of BBB tight junctions, ultimately increasing its permeability and potential accumulation in brain structures, including the choroid plexus (Bovio et al., 2021). Human biomonitoring data further support the potential role of Cd in neurodegeneration. Blood and urinary Cd levels have been consistently associated with AD-related mortality and with circulating or cerebrospinal fluid biomarkers linked to AD pathology (Min and Min, 2016; Peng et al., 2017; Schubert et al., 2023). Positive associations between Cd exposure and PD risk have also been reported (Lv et al., 2025). Adverse impacts of Cd on the human nervous system have been described at concentrations of > 0.8 μg/L (urine) and > 0.6 μg/L (blood) (Ruczaj and Brzoska, 2023). In general population surveys from industrialized countries, typical blood Cd concentrations range approximately from 0.004 to 7.0 μg/L, and urinary Cd from ∼0.03 to ∼14 μg/L, with higher values frequently observed among smokers or those with comorbidities (Ruczaj and Brzoska, 2023). Although typical biomonitoring concentrations are lower than those used *in vitro*, Cd persistence and bioaccumulation raise concern regarding long-term neuronal vulnerability.

Collectively, these findings demonstrate that, despite differences in intrinsic potency, the substances investigated significantly affect a neuronal model relevant for ND research, underscoring the need for further studies to elucidate their potential role in human neurodegeneration, especially under conditions of co-exposure.

### 4.2 Aflatoxin B1-containing binary mixtures present synergistic effects at low levels

A central finding of this work is the identification of synergistic interactions between AFB1 and ACR at low levels (Figure 4). Although ACR exhibited comparatively low cytotoxic potency in SH-SY5Y cells when tested alone, its toxicological impact increased markedly under mixture conditions. Notably, comparison of the IC_10_ for ACR alone versus in combination with AFB1 revealed a substantial shift in effective concentration, corresponding to a DRI of 4.4 under the CISNE model. This indicates that an almost 5 times lower concentration of ACR is required to achieve the same effect level in the presence of AFB1. Interestingly, the magnitude of dose reduction was proportionally greater for ACR than for AFB1 (DRI of 2.6), suggesting that the synergistic interaction was not equally driven by both contaminants. Rather, relatively low concentrations of AFB1 appeared sufficient to markedly enhance ACR-induced cytotoxicity. Although no mechanistic endpoints were experimentally evaluated in the present study, this asymmetric DRI profile may indicate that AFB1 acts as a sensitizing or modulatory component within the mixture, potentially lowering the threshold for ACR-induced toxicity. Such low-level synergism is particularly relevant in neurodegeneration, where repeated exposures may progressively compromise neuronal resilience. The observation that AFB1 amplifies the cytotoxic impact of a less toxic contaminant supports the concept that mycotoxins may act as modulators of mixture-driven neurotoxicity rather than merely independent toxic agents.

Unintentional dietary co-exposure to AFB1 and ACR is plausible in real-world settings. Mycotoxins frequently contaminate food crops worldwide, with more recent assessments indicating that as much as 60%–80% of crops may contain detectable levels of mycotoxins (Eskola et al., 2020). In the EFSA aflatoxin risk assessment, grains and grain-based products were the largest contributors to chronic identified as the major contributors to chronic dietary exposure to AFB1 across all age groups [EFSA Panel on Contaminants in the Food Chain (CONTAM) et al., 2020]. Cereals are also major dietary staples and are particularly susceptible to ACR formation during high-temperature processing at both industrial and household levels (Rifai and Saleh, 2020). EFSA identified soft bread, biscuits, crackers/crisp breads, breakfast cereals, processed cereal-based foods for infants and children, and coffee as important dietary sources of ACR, with cereal-based products being especially relevant for younger populations (EFSA CONTAM Panel, 2015). Similarly, the Irish Total Diet Study reported that cereals contributed 49% of adult and 47% of child dietary ACR exposure, while cereal-based products accounted for more than 80% of aflatoxin exposure in both adults and children (Food Safety Authority of Ireland, 2016). Although simultaneous analytical determination of AFB1 and ACR in the same food samples remains uncommon, these data support cereals and cereal-based foods as overlapping dietary exposure vehicles for both contaminants. Recent literature supports the relevance of combined assessment of ACR and mycotoxin effects in neuronal models (Bridgeman et al., 2024). While many studies have evaluated these contaminants separately, emerging evidence indicates that their combination enhances oxidative stress, apoptosis, and neurite impairment compared with individual treatments. For example, co-exposure of SH-SY5Y cells to ACR with penitrem A or 3-acetyldeoxynivalenol produced greater reductions in viability and more pronounced oxidative imbalance than single compounds, with ternary mixtures reducing viability by approximately 80% after 24 h (Bridgeman et al., 2023; Bridgeman et al., 2025).

Beyond ACR, AFB1 also influenced interaction profiles when combined with Cd^2+^. The AFB1/Cd^2+^ mixture produces additive to synergistic effects at low levels, depending on the mathematical model used to evaluate interactions (Figure 4). Considering AFB1/Cd^2+^ mixture ratio (2:1), the CISNE-derived IC10 corresponds approximately to 0.61 μM AFB1 and 0.30 μM Cd^2+^, which converts to about 190 μg/L AFB1 and 34 μg/L Cd^2+^. Whilst these concentrations are well above typical blood levels in the general population, they are comparable to other *in vitro* studies concentrations. Although these concentrations exceed typical circulating levels reported in the general population, they remain within the concentration ranges frequently employed in mechanistic *in vitro* neurotoxicity studies. Importantly, experimental evidence indicates that both contaminants can induce biologically relevant alterations at concentrations below overt cytotoxicity including concentrations close to or below those estimated for the IC_10_ mixture. For example, AFB1 has been associated with pro-inflammatory responses and blood–brain barrier dysfunction at low exposure levels, whereas Cd^2+^ has been reported to impair neurite development and dopaminergic neuronal function *in vitro* (Pak et al., 2014; Mehrzadet al., 2018; Park et al., 2020; Seguella et al., 2026). These observations support the relevance of evaluating low-effect interaction spaces when assessing mixture-driven neurotoxicity.

Occurrence data shows co-contamination of grains with mycotoxins and heavy metals is common. In one survey, all samples contained at least two contaminants and 77.3% contained more than four, with AFB1 and Cd detected in the same matrixes (Zhu et al., 2024). Yang et al. also reported frequent co-occurrence of multiclass contaminants, including mycotoxins and heavy metals, in these food matrices (Yang et al., 2020). Across Cd–mycotoxin combinations, additive or synergistic interactions are frequently reported (Fu et al., 2022). For example, Cd^2+^ combined with patulin or fumonisin B1 enhanced cytotoxicity and aggravated liver and kidney injury in both *in vitro* and *in vivo* models, supported by biochemical markers and histopathology (Cui et al., 2021; Liu et al., 2021). However, interaction outcomes are context-dependent. In the case of Cd^2+^ and deoxynivalenol, short-term or higher-dose exposures tended to favor synergistic responses, whereas longer or lower-dose conditions were more often associated with additive or antagonistic profiles (Guo et al., 2021; Fu et al., 2022). For AFB1 specifically, available *in vivo* data indicate additive toxicity following acute co-exposure with Cd^2+^, reflected in increased mortality, clinical alterations, and histopathological damage (Zhao et al., 2019).

Although interaction outcomes remain context-dependent, the present findings, together with available evidence, identify AFB1-containing mixtures as relevant drivers of combined neurotoxic stress under realistic exposure conditions, particularly at low effect levels pertinent to chronic neurodegenerative disease risk.

### 4.3 Acrylamide and cadmium exhibit antagonistic interactions at low effect levels

Both binary mixtures of ACR and cadmium as well as the ternary mixture including AFB1 exhibited antagonistic interactions at low effect levels.

ACR is known to interfere with cholinergic neurotransmission, including reduction in acetylcholinesterase (AChE) activity, thereby impairing acetylcholine turnover, contributing to neurological manifestations such as tremor, muscle weakness, and cognitive dysfunction (EFSA CONTAM Panel, 2015; Kopanska et al., 2022). On the other hand, some studies report Cd^2+^ exposure increases AChE activity in several brain regions, particularly striatum, hypothalamus, cerebellum, and at higher doses, hippocampus. This increase in AChE activity is consistent with enhanced acetylcholine degradation and reduced cholinergic neurotransmission, which the authors associate with the observed cognitive impairment (Goncalves et al., 2012). However, Cd-induced modulation of AChE remains inconsistent across studies and appears to depend on dose, exposure duration, and experimental model (Zarros et al., 2013). Given that undifferentiated SH-SY5Y cells express functional AChE (Onder et al., 2022), opposing modulation of this enzyme by ACR and Cd may result in counterbalancing effects on a shared target. Such compensatory regulation could attenuate overall cytotoxic responses and partially account for the antagonistic interactions observed in the mixtures. However, this interpretation remains speculative and would require direct enzymatic assessment.

In addition to cholinergic mechanisms, stress-response pathways may also contribute to the observed interaction pattern. Cadmium is a well-established inducer of metallothioneins (MTs) through metal-responsive regulatory mechanisms (Klaassen et al., 2009). MT isoforms are present in both neurons and glial cells, and chronic heavy metal exposure in rodent models has been associated with increased MT immunoreactivity in multiple brain regions, supporting their role as inducible defense proteins in the central nervous system (Navarro-Sempere et al., 2023). Acrylamide has also been shown to increase MT expression in non-neuronal tissues such as liver, likely as a component of electrophile-induced oxidative stress responses (Ghorbel et al., 2016). Moreover, co-exposure to aluminum and ACR significantly elevated total MT levels and MT1/MT2 gene expression in rat kidney compared with single exposures (Ghorbel et al., 2016). Although direct evidence for MT induction following combined ACR and Cd exposure in neuronal models is not available, concurrent activation of MT-dependent defense systems could enhance cellular resilience at low concentrations. Such adaptive responses may limit oxidative damage and metal reactivity, thereby reducing cell death relative to predictions based on simple additivity. The mechanistic considerations discussed are literature-based hypotheses intended to contextualize and provide a mechanistically plausible explanation for the antagonistic interactions observed at low effect levels in ACR-Cd^2+^ containing mixtures.

### 4.4 Concentration addition assumptions are violated by aflatoxin B1, acrylamide and Cd^2+^ concentration-response curves

Mixture effect predictions in risk assessment are commonly interpreted using two reference frameworks, CA and IA (Rodea-Palomares et al., 2015; Duarte and Vale, 2022). CA assumes similar modes of action, equal maximal efficacy, and parallel dose–response curves with constant relative potency across the full effect range. Although mixtures ratios were designed close to equipotency at IC_50_, this ratio holds only at that specific effect level. Because slopes differ, relative potency changes across the response range: at lower and higher fa values, the concentration ratios required to achieve equivalent effects increase. Classical CA does not account for this effect-dependent shift in potency, and predictions may be misinterpreted as synergism or subadditivity despite arising from model constraints (Grabovsky and Tallarida, 2004). For this reason, recent literature has questioned the suitability of CA-based approaches for chemically diverse mycotoxin mixtures (Kifer et al., 2020).

In the present dataset, AFB1 exhibited a Hill slope of 1.06, whereas ACR and Cd^2+^ presented Hill slopes of 1.41 and 0.91, respectively (Table 1). These differences indicate that relative potency changed across the response range, violating the constant relative potency assumption required for classical CA modeling. Mixture responses were therefore evaluated only against IA predictions (Figure 3). At low concentrations, which are most relevant for human exposure, IA predictions were generally close to the observed effects for ACR/AFB1, AFB1/Cd^2+^, and ACR/AFB1/Cd^2+^. In contrast, for the ACR/Cd^2+^ combination IA predicted stronger effects than those experimentally observed, resulting in an apparent antagonistic deviation. A key limitation of the IA framework is the strict assumption of complete independence between compounds, implying distinct mechanisms of action and non-overlapping targets. However, this assumption is often biologically unrealistic for toxicants. Chemicals with different primary mechanisms may converge on shared downstream pathways or cellular outcomes. Goldoni and Johansson reported that such convergence is common in toxicology, where compounds acting through distinct initiating mechanisms ultimately affect common cellular processes, complicating interpretation under strict independence assumptions (Goldoni and Johansson, 2007).

Mechanistic insight into mixtures composed of contaminants from different chemical classes remains limited. In such combinations, the primary molecular initiating events may differ substantially or remain insufficiently characterized, complicating mechanism-based predictions. Under these conditions, cell viability serves as an integrative endpoint that captures overall cellular survival and metabolic competence. Even when individual compounds act through distinct modes of action, a deviation from additivity at the level of viability demonstrates that the combined exposure elicits a net biological effect that cannot be inferred from single-compound responses alone. Accordingly, viability assays are appropriate for initial screening and prioritization of complex mixtures.

### 4.5 Quantifying interactions through combination-index approaches: CISNE is a modern alternative to the Chou-Talalay method

Originally developed for quantifying drug synergism, the Chou-Talalay approach has been widely used in the fields of ecotoxicology and toxicology to assess interactions among chemical pollutants or contaminants in mixtures (Chou, 2010; Alassane-Kpembi et al., 2017). Based on the MEE, expressed in dimensionless form, it enables the assessment of interactions between contaminants without requiring prior assumptions regarding potency equivalence or mechanistic similarity among mixture components (Chou, 2006).

Historically, estimation of MEE parameters relied on linearization of the equation, a strategy developed when nonlinear regression was computationally demanding. Although this transformation simplified parameter estimation, log-transformation alters the error structure of the data and introduces constraints on data processing ((Ashton, 2015), Supplementary Material of (Wooten and Albert, 2021)). In particular, responses must fall strictly between 0 and 1, requiring preprocessing steps such as normalization to control wells and removal or adjustment of values outside the admissible range. Trimming responses outside the theoretical bounds may remove biologically relevant variability and influence parameter precision (Boik et al., 2008). However, the original CT framework provides limited guidance on how experimental data should be processed prior to fitting (Chou, 2006).

In the present work, experimental variability occasionally produced Fa values slightly outside the theoretical interval. Instead of removing these observations, responses were constrained within the admissible range in order to preserve the full concentration spectrum. While this approach retains all measured effects, it imposes artificial bounds on the dataset and may influence parameter estimation under linearized MEE fitting. Another methodological consideration concerns replicates treatment. In examples presented by Chou (2006), a single response value is typically associated with each concentration, irrespective of the number of independent assays performed. In practice, however, experimental datasets frequently contain replicate-level variability that may influence model fitting. To explore the influence of data processing choices, we compared MEE parameters estimation using either mean responses or individual replicate data (Supplementary Table S1). As expected, fitting based on replicate values resulted in lower R^2^ values than fitting based on mean responses. From a statistical perspective, fitting based on mean values artificially increased apparent goodness-of-fit and replicate-level fitting therefore provides a more realistic representation of experimental uncertainty.

Recent approaches propose nonlinear implementations of the MEE as preferable alternatives to the classical linearized approach (Ashton, 2015; Watala et al., 2022). CISNE represents one such implementation, directly fitting the MEE through nonlinear regression without log transformation, reducing the risk of false positive or false negative classification of synergism at low or high effect levels (Garcia-Fuente et al., 2018). When comparing CT with CISNE goodness-of-fit in the same dataset (constrained CISNE versus CT), CISNE proves to fit better the data (Supplementary Table S1). Improved R^2^ values obtained after linearization do not necessarily justify the transformation, particularly when nonlinear regression is computationally accessible and avoids distortion of the error structure. Direct nonlinear fitting provides a more faithful representation of experimental data, especially at low and high effect levels where interaction classification is most sensitive. Both models have software available for the user with basic interfaces available, however available graphing and statistical platforms can perform the same calculations easily. In the present work, differences between CT and CISNE primarily influenced the strength of interaction classification rather than the overall toxicological interpretation (Figure 4). In general, CT tended to amplify deviations from additivity at low effect levels, predicting stronger synergism or antagonism compared with CISNE (Supplementary Table S2). Discrepancies were most evident for the AFB1/Cd^2+^ mixture in the low-dose region, where CT predicted synergistic effects at low Fa values whereas CISNE indicated additive to slightly antagonistic interactions. This distinction is particularly relevant in contaminant research, where low-level effects are most representative of realistic human exposure scenarios. In another dataset investigating the combined effects of platelet antagonists on ADP-induced platelet activation, CISNE also provided a better fit to the same experimental data than the CT model, as reflected by consistently higher R^2^ values. Importantly, the linear CT approach tended to predict stronger synergistic interactions, yielding lower CI values and larger synergy areas, whereas non-linear regression approaches produced more conservative and statistically robust estimates of interaction (Watala et al., 2022).

Although CT remains more familiar in the food toxicology research, the present results support broader adoption of CISNE for FC-mixture studies. Given that low-level effects are most relevant for chronic exposure scenarios implicated in neurodegenerative disease risk, careful consideration of modelling assumptions is essential to avoid overestimation or underestimation of mixture-driven neurotoxicity.

Despite the insights provided, some limitations should be acknowledged. First, the use of fixed equipotent mixture ratios does not reflect the variability of real-world contaminant exposures. Because dietary exposure to AFB1, ACR, and Cd varies with food matrix, processing conditions, and geographic setting, the present CI values should be interpreted as specific to the tested equipotent mixtures rather than as universal descriptors of combined toxicity. Broader designs based on multiple rays or full response surfaces, ideally anchored to occurrence or intake data, would strengthen external validity in future studies.

In addition, the study was limited to a single human-derived cell line, one exposure duration, and one cytotoxicity endpoint. Because the present study was based on cell viability and interaction modeling, it does not resolve the molecular mechanism of combined action. The data demonstrate outcome-level deviations from additivity, but they do not distinguish whether those deviations arise from convergent stress pathways, altered cellular uptake, toxicodynamic interactions, or other upstream events. For this reason, mechanistic interpretations in the present discussion should be considered literature-based hypotheses rather than experimentally established explanations for the observed synergy or antagonism. Therefore, future studies should incorporate additional endpoints and more complex biological models to better evaluate chronic low-dose exposure scenarios. Nevertheless, the present study enabled direct comparison of interaction models and identified AFB1 as a potential modulator of mixture-driven neurotoxicity.

## 5 Conclusion

This study demonstrated that mixture composition and mathematical modelling critically influence the interpretation of neurotoxic interactions among FCs in human SH-SY5Y neuronal cells. AFB1-containing mixtures exhibited the most relevant interaction patterns, highlighting that AFB1 may act as a modulator of combined neuronal stress under multi-contaminant exposure scenarios. In particular, at the lowest effect levels, which are the most relevant for human exposure, AFB1/ACR combination displayed synergism, whereas ACR/Cd^2+^ and the ternary mixture were predominantly antagonistic at the same effect level. The interaction profiles of AFB1/Cd^2+^ mixture were model-dependent (synergistic for CT and additive for CISNE), illustrating how different quantitative frameworks can yield divergent conclusions from the same dataset.

Methodologically, CISNE provided improved robustness with less data preprocessing compared with classical CT approach. Although both methods are grounded in the same theoretical framework, differences in regression strategy influence interaction classification, particularly at low effect levels most relevant to chronic exposure and neurotoxicity outcomes.

Collectively, these findings support the importance of mixture-based approaches in FC risk assessment and identify AFB1-containing mixtures as priorities for future mechanistic studies related to neurodegenerative disease risk.

## Data Availability

The original contributions presented in the study are included in the article/Supplementary Material, further inquiries can be directed to the corresponding authors.
