# Supplementary material for: Aflatoxin B1 modulates mixture-induced neurotoxicity under multi-contaminant exposure: insights from combination modeling in SH-SY5Y cells
Source: Front Pharmacol. 2026 Jul 3;17:1857857. doi: 10.3389/fphar.2026.1857857 (PMC13375712; doi:10.3389/fphar.2026.1857857)
Supplement: Supplementary file 1 [file Supplementaryfile1.docx]

Supplementary Material

# Supplementary Data


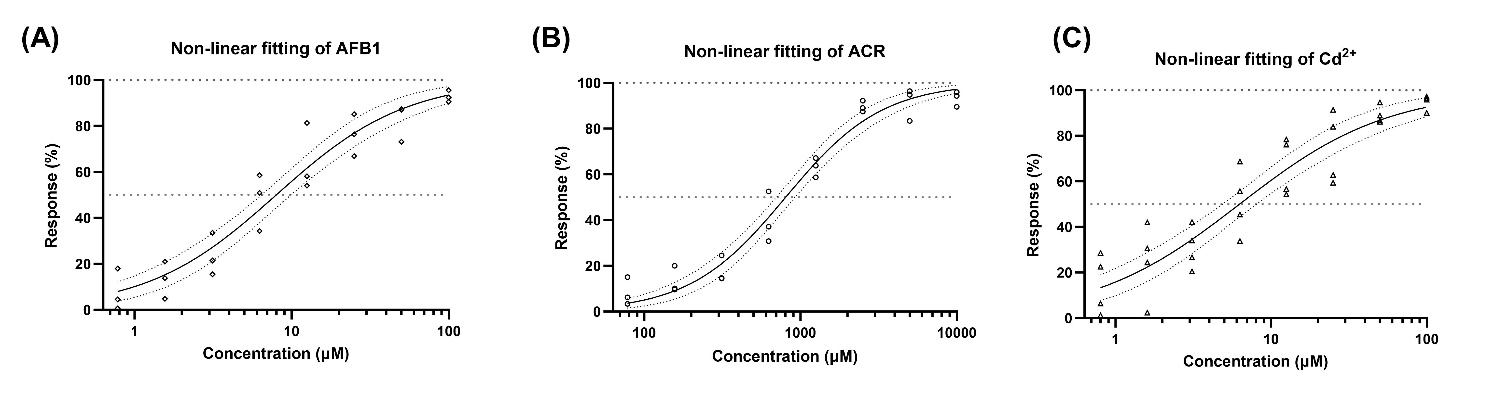


**Supplementary Figure 1.** Non-linear Hill model fits for single-compound concentration–response data in SH-SY5Y cells following 72 h exposure, assessed by the MTT assay: (A) aflatoxin B1 (AFB1), (B) acrylamide (ACR), and (C) Cd^2+^. Points represent individual experimental observations. Solid lines indicate the best-fit Hill curves, and dotted lines represent the 95% confidence bands. Horizontal dashed lines mark 50% response and 100% response.

**Supplementary Table 1.** Median-effect equation (MEE) parameter estimates for single contaminants and fixed-ratio mixtures in SH-SY5Y cells after 72 h exposure. For the Chou–Talalay approach, analyses were conducted using either mean response values or replicate data from different independent assays. Linearization parameters (X-intercept, Y-intercept ± SE, and linear correlation coefficient r) are reported together with the derived median-effect dose (Dm), slope (m), and the coefficient of determination (R²) obtained from the corresponding non-linear MEE fit using the linearization-derived parameters. For the CISNE implementation, Dm and m were estimated by direct non-linear regression using replicate data. When required, data was constrained (Fa bounded between 0 and 1) or unconstrained conditions, as indicated. Corresponding R² values reflect the goodness-of-fit of the non-linear model.

| **Contaminants** | **Model** | **Data input** | **Linearization Parameters** | **Dm**  **(95% CI)** | **m**  **(95% CI)** | **R^2^** |
| --- | --- | --- | --- | --- | --- | --- |
| **Individual Contaminants** | | | | | | |
| **AFB1** | **Chou-Talalay** | Mean data | X-intercept: 0.9356  Y-intercept: -0.9739 ±0.05053  r: 0.9949 | 8.62 | 1.04  *(0.94-1.15)* | 0.9898 |
|  |  | Replicate data | X-intercept: 1.0245  Y-intercept: -1.3572 ±0.21761  r: 0.8354 | 10.58 | 1.33  *(0.94-1.71)* | 0.6979 |
|  | **CISNE** | Mean | - | 7.96  *(6.75-9.41)* | 1.06  *(0.90-1.27)* | 0.9910 |
|  |  | Constrained replicate data | - | 7.96  *(6.44-9.88)* | 1.06  *(0.86-1.33)* | 0.9284 |
|  |  | Unconstrained replicate data | - | 8.71  *(6.74-11.31)* | 1.13  *(0.86-1.50)* | 0.9005 |
| **ACR** | **Chou-Talalay** | Mean data | X intercept: 2.878  Y intercept: -3.389 ± 0.2857  r: 0.9813 | 755.6 | 1.18  *(0.96-1.41)* | 0.9629 |
|  |  | Replicate data | X intercept: 2.880  Y intercept: -3.561 ± 0.2354  r: 0.9591 | 758.4 | 1.24  *(1.09-1.40)* | 0.9199 |
|  | **CISNE** | Mean data | - | 803.2  *(669.7-961.4)* | 1.41  *(1.11-1.83)* | 0.9884 |
|  |  | Replicate data | - | 803.2  *(700.6-919.7)* | 1.41  *(1.18-1.71)* | 0.9670 |
| **Cd^2+^** | **Chou-Talalay** | Mean data | X intercept: 0.7672  Y intercept: -0.7254 ± 0.05256  r: 0.9933 | 5.85 | 0.95  *(0.84-1.06)* | 0.9867 |
|  |  | Replicate data | X intercept: 0.81755  Y intercept: -0.8795 ± 0.1114  r: 0.9000 | 6.57 | 1.08  *(0.88-1.27)* | 0.8100 |
|  | **CISNE** | Mean data | - | 6.24  *(5.34-7.28)* | 0.91  *(0.79-1.04)* | 0.9921 |
|  |  | Replicate data | - | 6.24  *(4.84-8.00)* | 0.91  *(0.72-1.14)* | 0.8710 |
| **Mixtures** |  |  |  |  |  |  |
| **ACR/AFB1** | **Chou-Talalay** | Mean data | X intercept: 2.284  Y intercept: -2.886 ± 0.347  r: 0.9670 | 192.2 | 1.26  *(0.93-1.60)* | 0.9350 |
|  |  | Replicate data | X intercept: 2.305  Y intercept: -3.030 ± 0.242  r: 0.9474 | 201.8 | 1.32  *(1.12-1.51)* | 0.8976 |
|  | **CISNE** | Mean data | - | 261.2  *(197.3-342.4)* | 1.15  *(0.87-1.58)* | 0.9760 |
|  |  | Replicate data | - | 261.2  *(209.3-324.1)* | 1.15  *(0.92-1.47)* | 0.9266 |
| **AFB1/Cd^2+^** | **Chou-Talalay** | Mean data | X intercept: 1.008  Y intercept: -0.7229 ± 0.1134  r: 0.9326 | 10.18 | 0.72  *(0.44-0.99)* | 0.8697 |
|  |  | Replicate data | X intercept: 1.011  Y intercept: -0.7293 ± 0.0643  r: 0.9227 | 10.25 | 0.72  *(0.59-0.85)* | 0.8514 |
|  | **CISNE** | Mean data | - | 11.79  *(7.80-18.05)* | 0.86  *(0.56-1.32)* | 0.9376 |
|  |  | Replicate data | - | 11.79  *(9.60-14.50)* | 0.86  *(0.70-1.06)* | 0.9211 |
| **ACR/Cd^2+^** | **Chou-Talalay** | Mean data | X intercept: 2.94266  Y intercept: -7.0609 ± 0.6657  r: 0.9810 | 876.3 | 2.40  *(1.74-3.06)* | 0.9623 |
|  |  | Replicate data | X intercept: 2.9795  Y intercept: -8.7632± 0.7456  r: 0.94035 | 953.9 | 2.94  *(2.37-3.50)* | 0.8843 |
|  | **CISNE** | Mean data | - | 817.4  *(778.3-858.6)* | 2.59  *(2.32-2.92)* | 0.9991 |
|  |  | Constrained replicate data | - | 817.3  *(786.7-849.3)* | 2.59  *(2.37-2.84)* | 0.9963 |
|  |  | Unconstrained replicate data | - | *870.3*  *(793.5-957.6)* | *2.93*  *(2.35-3.85)* | *0.9786* |
| **ACR/AFB1/Cd^2+^ (160:2:1)** | **Chou-Talalay** | Mean data | X intercept: 2.49263  Y intercept: -4.0552 ± 0.30037  r: 0.9847 | 310.9 | 1.63  *(1.34-1.91)* | 0.9696 |
|  |  | Replicate data | X intercept: 2.58216  Y intercept: -5.1404 ± 0.45482  r: 0.8980 | 382.1 | 1.99  *(1.63-2.35)* | 0.8065 |
|  | **CISNE** | Mean Data | - | 394.0  *(350.8-442.0)* | 1.72  *(1.45-2.07)* | 0.9952 |
|  |  | Constrained replicate data | - | 394.0  *(1.53-1.95)* | 1.72  *(1.53-1.95)* | 0.9836 |
|  |  | Unconstrained replicate data | - | 464.9  *(1.77-2.73)* | 2.17  *(1.77-2.73)* | 0.9632 |

**Supplementary Table 2.** Combination Index (CI) values and interaction classification for binary and ternary mixtures calculated using the CISNE and Chou–Talalay models at increasing effect levels. Interpretation of CI values: very strong synergism (CI < 0.1); strong synergism (0.1 ≤ CI < 0.3); synergism (0.3 ≤ CI < 0.7); moderate synergism (0.7 ≤ CI < 0.85); slight synergism (0.85 ≤ CI < 0.9); nearly additive (0.9 ≤ CI ≤ 1.1); slight antagonism (1.1 < CI ≤ 1.20); moderate antagonism (1.20 < CI ≤ 1.45); antagonism (1.45 < CI ≤ 3.3); strong antagonism (3.3 < CI ≤ 10); very strong antagonism (CI > 10). Values are presented as mean CI with corresponding 95% confidence intervals. In the Chou–Talalay model, 95% CIs were obtained through sequential deletion analysis implemented in CompuSyn. In the CISNE model, 95% CIs were estimated using Monte Carlo simulations based on nonlinear regression uncertainty, providing probabilistic confidence bounds for CI estimates across effect levels.

|  |  | **AFB1/Cd^2+^** | |  | **ACR/AFB1** | |  | **ACR/Cd^2+^** | |  | **ACR/AFB1/Cd^2+^** | |
| --- | --- | --- | --- | --- | --- | --- | --- | --- | --- | --- | --- | --- |
|  |  | **CT model** | **CISNE model** |  | **CT model** | **CISNE model** |  | **CT model** | **CISNE model** |  | **CT model** | **CISNE model** |
| **Fa** |  | **CI (95% CI)** | |  | **CI (95% CI)** | |  | **CI (95% CI)** | |  | **CI (95% CI)** | |
| **0.05** |  | 0.30  (0.26-0.33) | 0.93  (0.26-1.60) |  | 0.67  (0.61-0.73) | 0.60  (0.18-1.01) |  | 10.10  (9.37-10.82) | 11.42  (4.45-18.38) |  | 3.96  (3.69-4.23) | 6.52  (3.27-977) |
| **0.10** |  | 0.43 (0.39–0.47) | 1.05 (0.47–1.62) |  | 0.63 (0.58–0.68) | 0.61 (0.29–0.94) |  | 6.80 (6.41–7.19) | 7.07 (3.95–10.19) |  | 3.01 (2.84–3.18) | 4.53 (2.85–6.22) |
| **0.25** |  | 0.73 (0.69–0.77) | 1.26 (0.84–1.68) |  | 0.58 (0.55–0.61) | 0.65 (0.44–0.86) |  | 3.82 (3.67–3.97) | 3.62 (2.74–4.51) |  | 2.02 (1.93–2.10) | 2.72 (2.12–3.32) |
| **0.50** |  | 1.25 (1.20–1.30) | 1.53 (1.17–1.89) |  | 0.53 (0.51–0.55) | 0.69 (0.54–0.84) |  | 2.15 (2.10–2.21) | 1.94 (1.65–2.23) |  | 1.35 (1.32–1.39) | 1.68 (1.43–1.93) |
| **0.75** |  | 2.15 (2.03–2.27) | 1.89 (1.20–2.58) |  | 0.49 (0.47–0.50) | 0.74 (0.52–0.96) |  | 1.22 (1.19–1.25) | 1.09 (0.87–1.31) |  | 0.91 (0.90–0.93) | 1.07 (0.85–1.28) |
| **0.90** |  | 3.70 (3.38–4.03) | 2.36 (0.93–3.80) |  | 0.45 (0.43–0.47) | 0.81 (0.43–1.18) |  | 0.69 (0.67–0.72) | 0.64 (0.44–0.84) |  | 0.62 (0.60–0.63) | 0.70 (0.48–0.92) |
